# A New Analytical Model to Estimate the Voltage Value and Position of the Pull-In Limit of a MEMS Cantilever

**DOI:** 10.3390/mi7040053

**Published:** 2016-03-24

**Authors:** Cevher Ak, Ali Yildiz

**Affiliations:** 1 Department of Electrical and Electronics Engineering, Toros University, Mersin 33140, Turkey; cevher.ak@toros.edu.tr; 2 Department of Electrical and Electronics Engineering, Mersin University, Mersin 33343, Turkey

**Keywords:** cantilever, electrostatic actuator, lumped model, pull-in limit, pull-in voltage, pivot model

## Abstract

In this study, a new analytical model is developed for an electrostatic Microelectromechanical System (MEMS) cantilever actuator to establish a relation between the displacement of its tip and the applied voltage. The proposed model defines the micro-cantilever as a rigid beam supported by a hinge at the fixed-end with a spring point force balancing the structure. The approach of the model is based on calculation of the electrostatic pressure centroid on the cantilever beam to localize the equivalent electrostatic point load. Principle outcome of the model is just one formula valid for all displacements ranging from the initial to the pull-in limit position. Our model also shows that the pull-in limit position of a cantilever is approximately 44% of the initial gap. This result agrees well with both simulation results and experimental measurements reported previously. The formula has been validated by comparing the results with former empirical studies. For displacements close to the pull-in limit, the percentage errors of the formula are within 1% when compared with real measurements carried out by previous studies. The formula also gives close results (less than 4%) when compared to simulation outcomes obtained by finite element analysis. In addition, the proposed formula measures up to numerical solutions obtained from several distributed models which demand recursive solutions in structural and electrostatic domains.

## 1. Introduction

Electrostatic cantilevers have been very popular due to their low-power requirements, small dimensions, and ease of fabrication. They have been used as a pressure sensor [1,2], as a microwave switch [3,4,5,6], as an air flow sensor [7,8,9], as an inkjet head [10,11,12], as chemical and biological sensors [13,14,15], and as an energy harvester for Microelectromechanical System (MEMS) devices [16,17,18].

**Figure 1:**
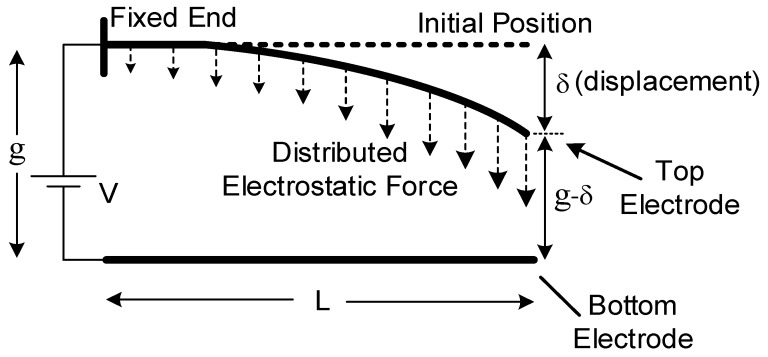
Side view of an electrostatic cantilever.

An electrostatic MEMS cantilever consist of two parallel beams forming a capacitor. The bottom beam is stationary and fabricated on the substrate. The top beam is suspended with an effective area A and is raised by a gap, *g*, over the ground beam. One side of the upper electrode is not fixed and entirely free to move and the opposite side is fixed to substrate. As voltage difference between conductive plates gets higher, the free-end will start to incline towards to bottom electrode due to electrostatic force. Actual behavior of a cantilever can be seen distinctly in Figure 1.

Derivation of an analytical formula to calculate the pull-in limit of a cantilever actuator is not a trivial task due to its non-linear behavior. Researchers have been using a lumped model for MEMS-based electrostatic cantilevers to estimate the pull-in limit position for more than two decades [19,20,21,22,23,24,25]. The lumped model approximates the pull-in limit position as one-third of the original spacing (*g*/3). Notwithstanding, previous research demonstrated experimentally that the pull-in limit goes beyond this approximation [26]. In order to present their distributed model approach, Hu *et al.* [26] employed a linearized governing equation for an actuator. Firstly, by using electric potential energy, kinetic and strain energy terms, the total energy formula was obtained. By putting this expression into Hamilton’s principle, a non-linear force term was achieved with a partial differential equation. Taylor series expansion of the force term under the assumption of small displacement to neglect the higher order terms were employed to obtain an electrostatic-structural coupling linear partial differential equation. This model yielded larger error percentages as the actuator tip was moving away from the starting post since small deflection was assumed and Taylor series was expanded around the initial position of the tip. When the free-end gets closer to the pull-in limit, errors as high as 10% are inevitable [26].

The Generalized Differential Quadrature Method (GDQM) has been employed by a later study. High-order polynomial approximation is used as an efficient and precise way to examine a linear vector space [27]. When the displacement is small, this approach also performs well with small error percentages. Similar to the previous method mentioned, when the tip gets away from the rest point, errors as high as 5% can be reached. A comparatively ordinary closed-form analytical approach model has been studied with reasonable errors in [28] as well. The model started with an empirical formula for the capacitance of a VLSI on-chip interconnect. Then, they derived a linear and uniform approximation to the non-linear electrostatic pressure. Additionally, the model has taken the electrostatic forces as a result of the fringing effect into account. Even though the model yields the pull-in voltage with reasonable errors, it does not present any information about the relation between the displacement of the tip of free electrode and the applied voltage. O’Brien *et al.* [29] calculated the capacitance value of the cantilever and derived a voltage statement which is also valid just for the pull-in limit case. They also fabricated MEMS cantilevers and conducted measurements for the pull-in voltage. Although their model showed a better approximation of the system, it still has some relatively large errors. Commonly used cantilevers have the matching width for both top and bottom electrodes. The cantilever devices fabricated in the study have the whole silicon wafer as a bottom electrode. When the structure is checked with software packages which utilize finite element method (FEM), it can be concluded that their model is just yielding small errors for cantilevers with wider bottom electrodes (CWBE). The same model cannot be used for the ordinary cantilever structures (OCS) which have the matching top and bottom electrode dimensions to get the similar error levels.

In this paper, a novel model was developed for an electrostatic micro-cantilever actuator. Unlike a widely-used lump model, which considers the zero-angle constraint and ignores the zero-displacement constraint of the fixed-end of the upper beam, our model takes the zero-displacement constraint into account and neglects the zero-angle constraint. A comparatively simple formula with a good approximation of the system was derived. The proposed formula is valid for all displacements which extent from initial to pull-in limit positions. The percentage errors of the formula are within 1% when compared to simulation results of various different cantilever structures for displacements close to the pull-in limit position. The proposed model also measures up to numerical results obtained from different distributed models while using lower computing efforts.

## 2. Lumped Model

**Figure 2:**
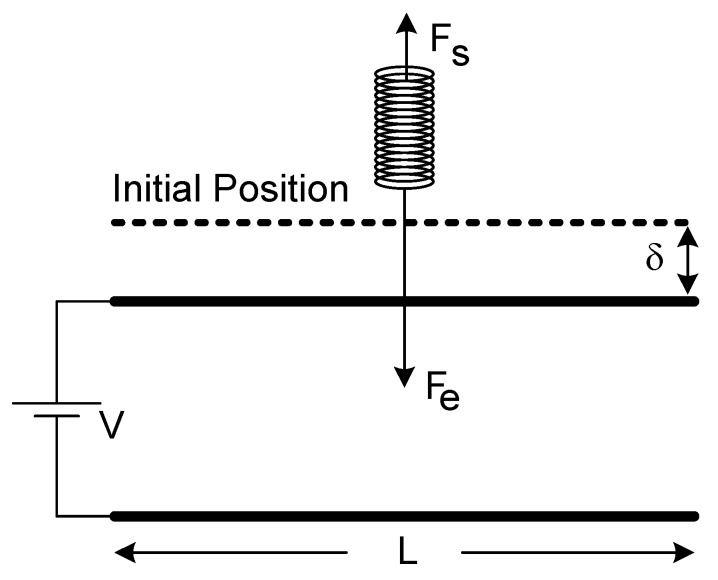
Sketch of the lumped model for a cantilever actuator.

A real actuator electrode system has two constraints: the fixed side of the upper beam has to hold both zero-angle and zero-displacement constraints. However, a lumped model takes only the first constraint into account for the sake of simplicity. As a result, the top beam moves vertically down as a whole structure, like a piston, whenever an electric potential difference is employed between the top and the bottom electrodes in the model. The top beam will return to its rest position owing to the restoring force of the bended beam as the potential difference is removed. This force is sometimes called as spring force, as well. The lumped model can be seen in Figure 2.

Figure 2 shows that zero-displacement constraint is neglected and, merely, the zero-angle constraint is reckoned in the lumped model. The model is quite simple to calculate the pull-in limit. Consequently, computations are simple and the pull-in limit can be calculated in a couple of steps as one-third of the original spacing between electrodes (*g*/3) [30]. However, by using software packages like COMSOL (Comsol, Inc., Burlington, MA, USA) and ANSYS (Ansys, Inc., Canonsburg, PA, USA), which utilize FEM, pull-in limit can be found as around 44% of the initial spacing. That value is in accord with the previous experimental studies [26,27]. Table 1 indicates certain simulation outcomes for various gaps between two electrodes for 2-D modeling. Results from these studies dictates that the lumped model is not suitable for a decent estimation of the cantilever system.

## 3. Pivot Model

**Figure 3:**
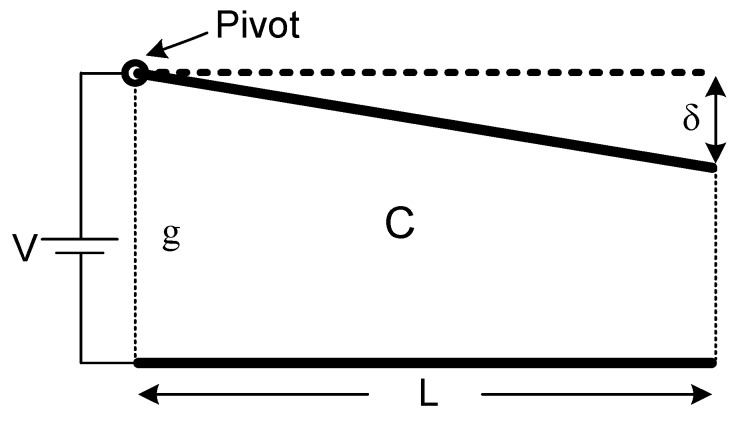
Pivot model for a cantilever actuator (not scaled).

Even though distributed models give a satisfying approximation of a cantilever actuator, they require more computing power. On the other hand, the lumped model simplifies the analytical calculations but fails to represent the system decently. We propose a new model named as the pivot model in order to get a good estimation of the system without requiring substantial computing power. In the proposed model, one end of the top beam is pivoted, so it is fixed to its original position, and the opposite end is entirely free to rotate linearly around this fixed pivoted end. Consequently, the top electrode was presumed as a non-flexible stick, like a rigid body. The non-bendable movement of top electrode can be examined clearly in Figure 3. These are reasonable assumptions when small angle bending is considered. The gap, *g*, between the electrodes is very small when compared to the length of electrodes, *L*, for a typical cantilever actuator structure used for extensively-preferred MEMS devices. Hereby, it is also reasonable to presume that *δ* << *L*.

Previously, we studied bisection model before the pivot model [31,32]. The model was dividing the top beam into two sections. The first section was free to rotate linearly around a point, named as the pivot, whose location was chosen by a trial and error method, and the second section was not moving at all and fastened to the fixed end. It was very successful, like the pivot model, when compared to simulation outcomes and earlier experimental results carried out by other researchers. However, that model was a little bit complex when compared to the current model.

We also proposed an inversely-designed model for fixed-fixed electrostatic actuators [33]. In this case, the structure was divided into three sections symmetrically by four pivot points, two of them are at the ends of the upper electrode and the other two were chosen by a trial and error method again. The model can estimate the pull-in limit position successfully and percentage errors of the model are within 2% when compared with simulation results.

The pivot model gives not only a simple analytical model but also a very good approximation of a cantilever system. There is no displacement at the pivoted side of the upper electrode. Thus, the model only takes the zero-displacement constraint into account and omits the zero-angle constraint. In this instance, calculations are a little arduous, yet straightforward. Fringing field capacitances due to beam width and beam thickness are not included, and only the parallel plate capacitance value is considered in the model for the sake of simplicity. Hence, for a *δ* displacement from the rest position of the upper electrode, capacitor value of the system can be computed as:

(1) $$C=\frac{\varepsilon_{0}wL}{\delta }\text{ln}\left(\frac{g}{g-\delta }\right)$$

where *ε*_0_ and *w* are permittivity of free space and width of the cantilever, respectively. Details of the capacitance calculation can be found in [31,32]. So, the electrostatic force becomes:

(2) $$F_{e}=\frac{\varepsilon_{0}wLV^{2}}{2}\left[\frac{\delta +\left(g-\delta \right)\text{ln}\left(\frac{g-\delta }{g}\right)}{\delta^{2}\left(g-\delta \right)}\right]$$

**Figure 4:**
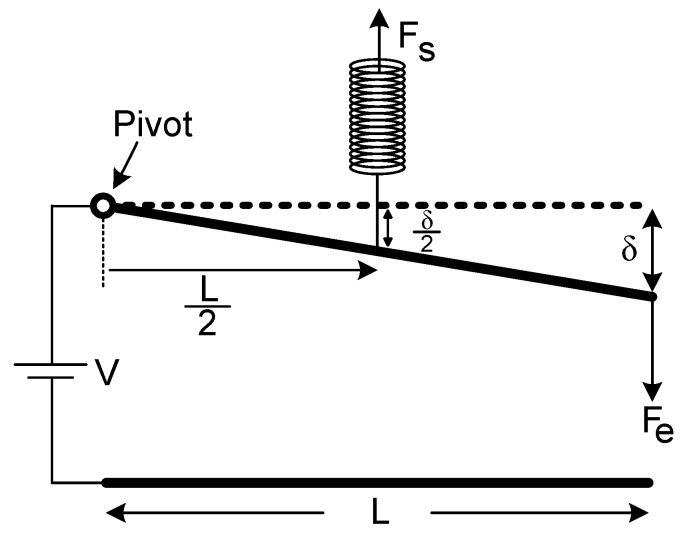
Representative equivalent forces were sited arbitrarily.

However, this force term gives the total electrostatic force which is distributed and non-uniform throughout the upper electrode surface. It is very troublesome to demonstrate it in a plain formula. Hence, it can be replaced with a representative equivalent single-force term. In order to find the position of the pull-in limit, the representative equivalent single-force term can be temporarily located at a random point, initially, since the location of the force will not shift the pull-in limit position. Consequently, both electrostatic and restoring force terms were placed arbitrarily, like in Figure 4.

Their exact locations are going to be calculated in later steps to establish a relation between the voltage difference and the maximum displacement of the upper electrode. The restoring force is:

(3) $$F_{s}=\frac{\delta }{2}k$$

where *k* is elastic constant of the spring and *δ* is the displacement of the free tip of upper electrode. Since the system is at equilibrium, the overall moment has to be zero around the pivot point. Thus, for small-angle approximation, it can be written by using Equations (2) and (3) as:

(4) $$\frac{\delta }{2}k=\varepsilon_{0}wL\left[\frac{\delta -\text{ln}\left(\frac{g}{g-\delta }\right)\left(g-\delta \right)}{\delta^{2}\left(g-\delta \right)}\right]V^{2}$$

and potential difference term can be get from Equation (4) as:

(5) $$V={\left[\frac{k\delta^{3}\left(g-\delta \right)}{2\varepsilon_{0}wL\left(\delta -\text{ln}\left(\frac{g}{g-\delta }\right)\left(g-\delta \right)\right)}\right]}^{\frac{1}{2}}$$

The position is named as the pull-in limit and can be found when the derivative of the potential difference is taken with respect to *δ* and is equated to zero [21]. The upper electrode will collapse towards onto bottom electrode after this point. Therefore:

(6) $$\frac{dV}{d\delta }=-\sqrt{\frac{k\delta }{2\varepsilon_{0}wL\left(g-\delta \right)}}\left[\frac{4\delta^{2}-3g\delta +\left(3\delta^{2}-6g\delta +3g^{2}\right)\text{ln}\left(\frac{g}{g-\delta }\right)}{{\left[\delta -\text{ln}\left(\frac{g}{g-\delta }\right)\left(g-\delta \right)\right]}^{\frac{3}{2}}}\right]=0$$

Getting an analytical solution to Equation (6) is inconvenient. Hence, a computational numerical solution has been achieved as:

(7) $$\delta_{\text{MAX}}=0.4404g$$

This outcome is well-matched to the measured values given in an experimental study [26] and from ANSYS and COMSOL simulations which are shown in Table 1.

If we chose the system forces at different locations at the beginning, we would have exactly the same result since only the term in square root would be changed which is not affecting the root of Equation (6). Although arbitrary positioning of the system forces gives us a correct value of the pull-in limit, it cannot establish any relation between the displacement and the voltage difference between the electrodes. Thus, the system forces have to be emplaced at their exact locations.

## 4. Positioning the Forces

**Figure 5:**
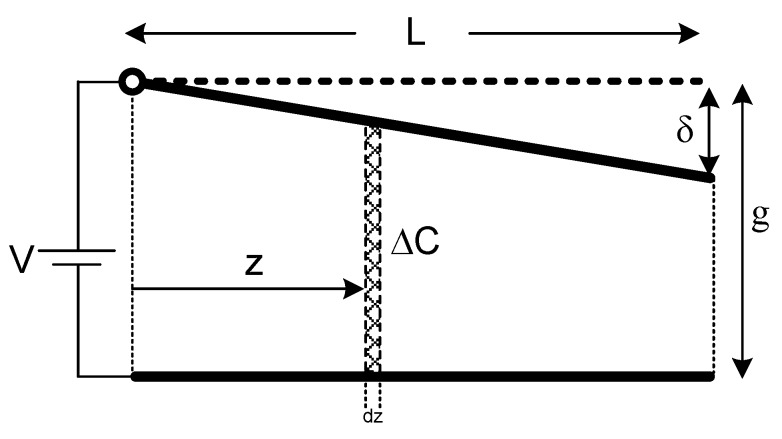
Calculation of total electrostatic moment.

In order to find the position of representative single equivalent electrostatic force, the total electrostatic moment of the system has to be divided by the total electrostatic force. The total electrostatic moment of the system can be calculated as seen in Figure 5.

Infinitesimal capacitance term equals to:

(8) $$\Delta C=\frac{\varepsilon_{0}wLdz}{gL-\delta z}$$

Then, the infinitesimal electrostatic force term will be:

(9) $$dF_{e}=\frac{1}{2}\frac{d(dC)}{d\delta }V^{2}\;\;=-\frac{1}{2}\frac{d}{d\delta }\left(\frac{\varepsilon_{o}wLdz}{gL-\delta z}\right)V^{2}\;=\frac{\varepsilon_{o}wLV^{2}}{2}\frac{zdz}{{\left(gL-\delta z\right)}^{2}}$$

Hence, the infinitesimal electrostatic moment term can be easily acquired by multiplying the infinitesimal electrostatic force by the distance to the pivot point, which is also named as the moment arm (*z_e_*). Here, small angle approximation is assumed again. So, the infinitesimal electrostatic force and moment arm can be presumed to be perpendicular to each other.

(10) $$dM_{e}=dF_{e}z_{e}=\frac{\varepsilon_{o}wLV^{2}}{2}\frac{z^{2}dz}{{\left(gL-\delta z\right)}^{2}}$$

In order to calculate total electrostatic moment of the structure, one needs to take the integral through the whole length of the upper beam:

(11) $$M_{e}=\frac{\varepsilon_{o}wLV^{2}}{2}\overset{L}{\underset{z=0}{\int }}\frac{z^{2}dz}{{\left(gL-\delta z\right)}^{2}}\;\;=\frac{\varepsilon_{o}wL^{2}V^{2}}{2}\left[\frac{2g\delta -\delta^{2}+2g\left(g-\delta \right)\text{ln}\left(\frac{g-\delta }{g}\right)}{\delta^{3}\left(g-\delta \right)}\right]$$

The total distributed electrostatic moment was calculated from distributed electrostatic force terms. When a distributed electrostatic force term is replaced by a representative equivalent single force term, the total moment can also be written as:

(12) $$M_{e}=F_{e}z_{e}$$

where *z_e_* is the position of replacement of single electrostatic force *F_e_*. When Equations (11) and (12) are solved simultaneously, we get:

(13) $$z_{e}=\frac{L\left[2g\delta -\delta^{2}+2g\left(g-\delta \right)\text{ln}\left(\frac{g-\delta }{g}\right)\right]}{\delta \left[\delta +\left(g-\delta \right)\text{ln}\left(\frac{g-\delta }{g}\right)\right]}$$

At the pull-in limit case, *δ* = 0.4404*g*. When *δ* is substituted in Equation (13), *z_e_* can be obtained via:

(14) $$z_{e}=0.73L$$

The stiffness restoring force is, similarly, a distributed force. Likewise, it can be replaced with a single equivalent restoring force, as well. By using a small angle assumption, the spring force term can be found via:

(15) $$F_{s}=k\delta$$

where *k* is stiffness constant which can be obtained for a cantilever beam as [34]:

(16) $$k=\frac{2}{3}Ew{\left(\frac{t}{L}\right)}^{3}$$

Since the cantilever actuator is at equilibrium, net torque about the pivot point has to be zero. Therefore, the electrostatic moment term has to be equal to restoring term.

(17) $$z_{s}F_{s}=0.73LF_{e}$$

where *z_s_* is the position of replacement single restoring force. Thus, the location of single equivalent restoring force can be found numerically:

(18) $$z_{s}=0.62L$$

**Figure 6:**
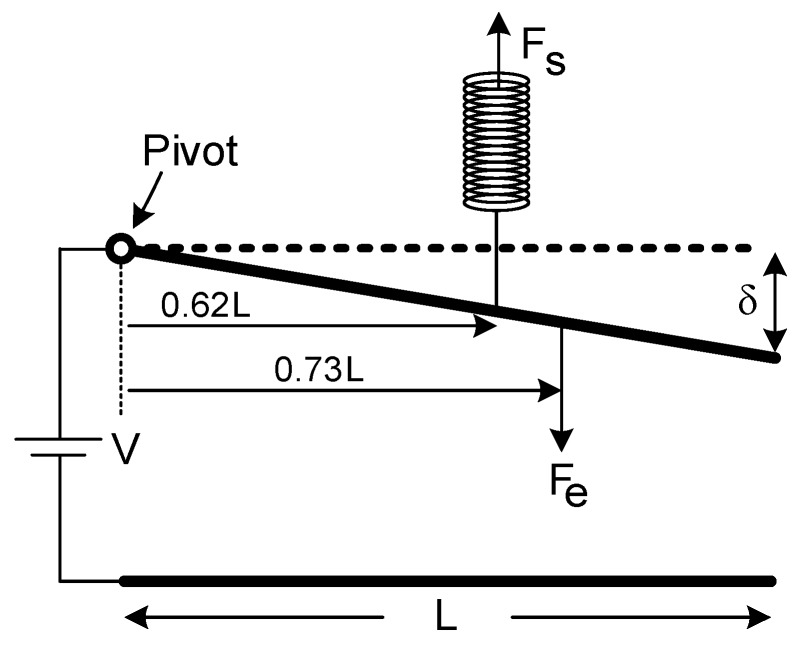
Repositioning the representative system forces at exact locations as a final Pivot Model.

Final semblance of the pivot model can be seen in Figure 6 with representative equivalent forces were put to their exact places. At the beginning of the model, these forces were placed intuitively. Therefore, it could not establish a good connection between the displacement of the free-end of the upper electrode and the applied voltage since the locations of the forces were not exact.

If we repeat the steps in the previous section and use Equation (16) in order to find an accurate *V* value after the forces are placed to their exact locations, we get:

(19) $$V=\frac{21}{25}{\left[\frac{Et^{3}\delta^{3}\left(g-\delta \right)}{\varepsilon_{0}L^{4}\left(\delta -\text{ln}\left(\frac{g}{g-\delta }\right)\left(g-\delta \right)\right)}\right]}^{\frac{1}{2}}$$

where *E* is the Young’s modulus of the structural material of the upper electrode. As it can be seen from Equations (5) and (19), only the constants are different. This voltage is valid for the whole displacement values ranging from the initial to the pull-in limit positions. In order to find a direct pull-in voltage formula, the pull-in displacement value of *δ* in Equation (7) should be inserted into Equation (19).

(20) $$V_{\text{Pull-in Limit}}=\frac{27}{50}{\left[\frac{Et^{3}g^{3}}{\varepsilon_{0}L^{4}}\right]}^{\frac{1}{2}}$$

Only Equation (20) is used to compare with the simulation outcomes since previous studies only offer a value for the pull-in voltage. For example, Chowdhury *et al.* [28] does not present any information about the applied voltage and the displacement of the tip of upper electrode. O’Brien *et al.* [29] showed a better approximation of the system, and still have some comparatively larger errors. When our pull-in voltage formula in Equation (20) is compared with their measurements, the pivot model has higher error, as seen in Table 2. Nonetheless, their fabricated cantilevers have a wider bottom electrode than the top one. Ordinary cantilevers have the same width for both the top and bottom electrodes. When their structure is checked with COMSOL software, it can be assured that their model is giving small errors for cantilevers with wider bottom electrodes (CWBE). However, the same model cannot be used with the similar error levels for ordinary cantilever structures (OCS). On the other hand, the pivot model shows exceptionally accurate results (less than 4%) as seen in Table 3 when compared to the COMSOL simulation for OCS MEMS devices.

It can be easily seen from Table 2 and Table 3 that their empirical result is compatible with the CWBE simulation rather than OCS simulation outcome since they fabricated the cantilevers with a wider bottom electrode structure. Since the bottom electrode is very wide, a fringing effect plays an important role in getting a smaller pull-in voltage value when compared with OCS.

Polysilicon is the material typically used for manufacturing micro-fabricated devices due to its ease of fabrication and good performance on micro-devices [35,36]. The average value of Young’s modulus of polysilicon which is deposited by low pressure chemical vapor deposition (LPCVD) method was measured as 170 GPa [37] and this value is used in all simulations demonstrated in this paper, except in Table 2 and Table 3.

Table 4 and Table 5 demonstrate the comparison of ANSYS and COMSOL simulation outcomes and results calculated from the pivot model (from Equation (19)) and percentage errors. All percentage errors are less than 3.5% when compared with both ANSYS and COMSOL results.

Table 6 shows comparison of previous experimental results, distributed model results [26,27], pivot model outcomes, and percent errors, with respect to previous experimental measurements. Percentage errors gets even better (less than 2%) for real measurements. They also reported that the pull-in voltage is at a level of 68.5 V. When Equation (20) is used, that value can be found as 68.1 V, which is a remarkably close value with an error of 0.6%. Since the pivot model is utilized at the pull-in limit case, the percentage error becomes smaller when getting closer to the pull-in limit which can be evidently seen in Table 6. To make the comparison more clear, ANSYS simulation outcomes are also appended.

## 5. Discussion and Conclusions

In this work, a model which gives the pull-in limit displacement for a cantilever actuator around 44% of the initial gap is presented. It achieves a significant consistency with results from software (ANSYS and COMSOL) simulations, distributed model [26], and previous experimental measurements [26,27]. In addition, the proposed model demonstrates a good connection between the voltage difference and the displacements extent from the initial to the pull-in limit positions. This is a contribution where some previous studies [28,29] lack. The pivot model delivers values which are conformable to those computed from FEM software (ANSYS and COMSOL) simulations. All of the percentage errors are within 4%, the largest percentage error is 3.78% and it can be seen in Table 3. When compared with lumped model, pivot model delivers an improved value for the pull-in limit. Additionally, in comparison to former distributed models which give relatively small errors with more computational power, the pivot model delivers an uncomplicated formula which gives small errors with a little computational capacity. Furthermore, the model gives satisfying estimations for the applied voltage for desired displacements. When compared to previous experimental measurement results, the pivot model can be found to be substantially successful, as well. Percentage errors of the pivot model are comparable when the displacement is close to the rest position, and delivers even better results when the displacement comes closer to the pull-in limit. The most prominent advantage of the pivot model is delivering a satisfying estimation of the system with a simple formula. Demanded voltage difference for both desired displacement and pull-in limit position can be calculated effortlessly rather than utilizing numerical distributed methods, which are time consuming and require more computing power.

Although the pivot model is remarkably accurate, particularly around the pull-in limit position, it has some limitations since it neglects some physical constraints for the sake of getting an effortless formula. Firstly, the fringing effect was not taken into account in the proposed model. If two parallel plates forming a capacitor, like in the cantilever case, the electric field does not end abruptly at the boundary of the beams. There are some fields outside the electrodes that curve from one beam to the other and this induces the real capacitance to be different than what it is calculated ideally.

Secondly, the model also assumes that the upper beam is a rigid body. Hence, the length of the electrode is constant and non-bending even at the boundary of the pull-in limit position. Therefore, it also assumes that the formation of both transverse and axial stresses would not be possible even in the full bending situation.

Thirdly, the formula is successful for exclusively-long electrodes where (*L* ≥ 5*w*) since the model ignores the shear stress near the fixed end of the upper electrode. In addition, the model is successful for wide beams (*w* ≥ 5*t*) [28,38]. This can be clearly seen in Table 4 and Table 5.

Fourthly, the formula delivers very small errors for the ordinary cantilever structures (OCS) which have matching top and bottom electrode dimensions. However, it is not successful for cantilevers with wider bottom electrodes (CWBE).

Lastly, the model disregards any atmospheric loading on the upper beam. Hence, the cantilever operates in vacuum.

## Figures and Tables

**Table 1:** ANSYS and COMSOL simulation of pull-in outcomes for a cantilever with *L* = 300 µm.

Initial Gap (µm)	ANSYS Pull-In Gap (µm)	ANSYS Pull-In Gap/Initial Gap	COMSOL Pull-In Gap (µm)	COMSOL Pull-In Gap/Initial Gap
2	0.881	0.4405	0.884	0.4420
4	1.761	0.4403	1.769	0.4422
5	2.202	0.4404	2.212	0.4424
10	4.403	0.4403	4.424	0.4424
20	8.809	0.4405	8.848	0.4424

Width, thickness, and material properties of cantilever do not affect the pull-in limit. They affect only the applied voltage to reach the pull-in limit (pull-in voltage).

**Table 2:** Comparison of *V*_max_ (pull-in voltage) values of a previous empirical study [29], pivot model, and COMSOL result for a cantilever with a wider bottom electrode (CWBE).

COMSOL for CWBE	Empirical *V*_max_ (V) [29]	*V*_max_ (V) [29]/% Error (CWBE)/% Error (Empirical Result)	Pivot Model/% Error (CWBE)/% Error (Empirical Result)
18.30	17.60	19.18/4.81/8.98	21.97/20.05/24.83

Length = 160 µm, initial gap = 2 µm, width = 6 µm, thickness = 2 µm, *E* = 150 GPa.

**Table 3:** Comparison of *V*_max_ (pull-in voltage) values of a previous empirical study [29], pivot model, and COMSOL result for an Ordinary Cantilever Structure (OCS).

COMSOL	*V*_max_ (V) [29]/% Error	Pivot Model/% Error
21.17	19.18/9.40	21.97/3.78

Length = 160 µm, initial gap = 2 µm, width = 6 µm, thickness = 2 µm, *E* = 150 GPa.

**Table 4:** Comparison of *V*_max_ (pull-in voltage) values for various lengths.

Cantilever Length (µm)	V_max_ (V) Pivot Model	V_max_ (V) (ANSYS)	% Error of the Model with Respect to ANSYS	V_max_ (V) (COMSOL)	% Error of the Model with Respect to COMSOL
150	26.619	27.341	2.712	27.070	1.694
200	14.973	15.418	2.972	15.240	1.783
250	9.583	9.899	3.298	9.760	1.847
300	6.655	6.828	2.600	6.780	1.878
400	3.743	3.860	3.126	3.820	2.057
500	2.396	2.472	3.172	2.450	2.254

Initial Gap = 2 µm, width = 50 µm, thickness = 2 µm, E = 170 GPa.

**Table 5:** Applied voltage differences for various displacements of the free-end of the upper electrode.

Displacement (µm) and (*δ*/*g*)	Voltage (V) Pivot Model	Voltage (V) (ANSYS)	% Error of the Model with Respect to ANSYS	Voltage (V) (COMSOL)	% Error of the Model with Respect to COMSOL
0.05829 (2.91%)	9.797	10.0	2.075	9.85	0.544
0.1386 (6.93%)	14.689	15.0	2.119	14.78	0.621
0.2714 (13.57%)	19.574	20.0	2.178	19.72	0.747
0.5165 (25.83%)	24.431	25.0	2.328	24.68	1.018
0.6028 (30.14%)	25.387	26.0	2.414	25.70	1.233
0.7419 (37.10%)	26.324	27.0	2.566	26.69	1.389
0.7654 (38.27%)	26.417	27.1	2.586	26.80	1.450
0.7963 (39.82%)	26.512	27.2	2.597	26.91	1.503
0.8146 (40.73%)	26.553	27.25	2.624	26.97	1.569
0.8808 (44.04%)	26.619	27.341	2.712	27.07	1.694

Length = 150 µm, initial gap = 2 µm, width = 50 µm, thickness = 2 µm, E = 170 GPa.

**Table 6:** Comparison of end gap displacement for different voltage values.

Voltage (V)	Experimental (µm) [26]	Distributed Model (µm) [26]/(Error)	GDQM (µm) [27]/(Error)	Pivot Model (µm)/(Error)	ANSYS (µm)
20	90.5	90.2/(0.3%)	90.2/(0.3%)	90.3/(0.2%)	90.4
40	84.6	84.3/(0.4%)	84.1/(0.6%)	84.7/(0.1%)	85.1
60	70.0	71.5/(2.1%)	69.1/(1.3%)	71.3/(1.9%)	73.2
65	64.0	67.2/(5.0%)	59.6/(6.9%)	64.1/(0.2%)	67.6
67	59.0	65.0/(10.2%)	-	59.1/(0.2%)	64.5

Errors are with respect to experimental results. *E* = 156 GPa, *L* = 20 mm, *w* = 5 mm, *t* = 57 µm, *g* = 92 µm.
